# Integrated non-targeted and targeted metabolomics reveals tryptophan metabolism disorders in hypertension

**DOI:** 10.3389/fcvm.2026.1805381

**Published:** 2026-08-05

**Authors:** Jing-wen Yue, Yun-feng Fang, Li Jiang, Tong-shuai Wang, Zhen-xian Zhang

**Affiliations:** 1 Traditional Medicine Department, Yueyang Hospital of Integrated Traditional Chinese and Western Medicine, Shanghai University of Traditional Chinese Medicine, Shanghai, China; 2 Institute of Cardiovascular Diseases, Tongren Hospital, Shanghai Jiao Tong University School of Medicine, Shanghai, China; 3 Hongqiao International Institute of Medicine, Shanghai Tongren Hospital, Shanghai Jiao Tong University School of Medicine, Shanghai, China; 4 School of Public Health, Shanghai Jiao Tong University School of Medicine, Shanghai, China

**Keywords:** hypertension, kynurenine pathway, metabolomics, network pharmacology, serotonin pathway, tryptophan metabolism

## Abstract

**Objective:**

This study aimed to characterize serum metabolic profiles in hypertensive patients and investigate the regulatory role of tryptophan metabolism.

**Methods:**

We employed an integrated metabolomics strategy that combined non-targeted screening with targeted validation. From November 2022 to November 2023, we enrolled 25 newly diagnosed, treatment-naïve hypertensive patients and 25 age- and sex-matched healthy controls (1:1 ratio) from a local Shanghai hospital, together with their clinical baseline data. Non-targeted metabolomics, combined with Principal Component Analysis (PCA) and Orthogonal Partial Least Squares Discriminant Analysis (OPLS-DA) were used to identify differential metabolic pathways. Subsequent targeted metabolomics analysis was conducted to quantify key molecules involved in the tryptophan metabolic pathway.

**Results:**

Non-targeted metabolomics revealed 2,931 metabolites, with 759 showing differential expression (296 up-regulated and 463 down-regulated), emphasizing tryptophan metabolism as the central dysfunctional pathway. Targeted validation confirmed abnormalities in three branches of tryptophan metabolism: 5-hydroxytryptamine (5-HT) was down-regulated by 30% (Fold change = 0.69, *P* = 0.002), while five metabolites, such as 5-HT acid (Fold change = 1.33, *P* = 0.002) and kynurenine (Fold change = 1.35, *P* = 0.004), exhibited significant up-regulation (all *P* < 0.05). Network pharmacology analysis indicated strong binding affinities between tryptamine metabolites (Serotonin and L-5-Hydroxytryptophan), kynurenine metabolites (3-Hydroxykynurenine and Kynurenine), and indole metabolites (Hydroxyindoleacetic acid and Indolelactic acid) with their respective targets SLC6A4, KYNU, and SLC6A2.

**Conclusion:**

Dysregulation of the tryptophan metabolic network-characterized by kynurenine pathway activation, 5-HT pathway inhibition, and microbial-indole pathway imbalance-is a key pathological mechanism in hypertension. These key molecules regulate targets such as SLC6A4, KYNU, and SLC6A2, potentially offering mechanistic insights for future precision intervention studies.

## Introduction

Hypertension is a major risk factor for cardiovascular disease, and its global burden continues to rise. According to the World Health Organization (WHO) Global Report on Hypertension 2023, 1.3 billion adults are affected (1). In China (2021–2022), the awareness, treatment, and control rates among adults were 43.3%, 38.7%, and 12.9%, respectively, indicating a critical public health challenge (2). Hypertension is not a monolithic condition as etiologies and pathogenic mechanisms vary considerably among individuals. Beyond traditional risk factors, emerging factors such as gut microbiota dysbiosis, sleep disorders, and epigenetic regulation of the renin-angiotensin-aldosterone system (RAAS) have become research focal points (3–6). Tryptophan metabolism, which integrates immune regulation, neurotransmitter balance, and gut-microbiota interactions, serves as a pivotal entry point for deciphering the heterogeneity of hypertension (7). Thus, exploring novel pathophysiological mechanisms is essential for advancing precision prevention and treatment.

Historically, hypertension research has focused predominantly on hemodynamic parameters and the RAAS. However, emerging omics data suggests that hypertension is a systemic syndrome characterized by profound metabolic remodeling and alterations in biochemical fluxes (7). Factors such as chronic low-grade inflammation, gut microbiota dysbiosis, and epigenetic modifications are now recognized as essential contributors to blood pressure elevation. Metabolomics, a core component of systems biology, provides an indispensable tool for identifying these subtle metabolic perturbations (8, 9). By assessing the end products of genomic, proteomic, and environmental processes, metabolomics bridges the gap between clinical manifestations and underlying molecular mechanisms, thereby facilitating the shift towards precision cardiovascular medicine.

Tryptophan (Trp), an essential amino acid, is a precursor for bioactive compounds that regulate vascular tone, immune responses, and neuroendocrine balance (10–12). Approximately 95% of dietary tryptophan is metabolized via the Kynurenine pathway (KP), which is highly sensitive to inflammatory stimuli mediated by enzymes like indoleamine 2,3-dioxygenase (IDO1). The remaining tryptophan is metabolized via the serotonin (5-HT) pathway, which is crucial for vascular reactivity, and the microbial-indole pathway, essential for intestinal homeostasis (10, 13). Dysregulation of these pathways has been associated with various cardiovascular pathologies, underscoring the need for a comprehensive understanding of tryptophan metabolism in hypertension that integrates clinical data with molecular mechanisms.

Prior investigations have often been limited by focusing on isolated components or single catabolic branches. Therefore, this study adopted an integrated screening–verification–bioinformatics strategy. We first identified core abnormal pathways via non-targeted metabolomics, quantified key molecules through targeted analysis, and predicted potential targets using network pharmacology and molecular docking. Our goal was to map the hypertension-specific tryptophan metabolic landscape and identify potential metabolic biomarkers to advance diagnostic research in hypertension.

## Materials and methods

### Study design and participant stratification

This investigation utilized a case-control design. A total of 58 subjects were initially screened from Shanghai Tongren Hospital between November 2022 and November 2023. To approximate the real-world onset pattern of hypertension, we selected newly diagnosed cases from treatment-naïve patients within the age range of highest prevalence who had no history of taking medications that could confound metabolomic analyses. All hypertensive participants were diagnosed according to the *Chinese Guidelines for the Prevention and Treatment of Hypertension* (2024), requiring consistent blood pressure readings exceeding 140/90 mmHg. To minimize confounding effects on the metabolomic analyses, we also rigorously applied the exclusion criteria listed below: (1) secondary hypertension; (2) congenital heart disease; (3) other diseases causing left ventricular hypertrophy (e.g., aortic stenosis, hypertrophic cardiomyopathy, aortic regurgitation, etc.); (4) severe hepatic or renal dysfunction; (5) chronic obstructive pulmonary disease; (6) malignant tumors; and (7) pregnancy. Following rigorous clinical evaluation, 25 healthy controls were selected via 1:1 matching based on age, and sex. The study was conducted in accordance with the Declaration of Helsinki and received formal approval from the Institutional Review Board of Shanghai Tongren Hospital (Approval No. K2024-059-01).

Additionally, detailed demographic data, smoking history, and alcohol consumption patterns were recorded during face-to-face interviews. All participants underwent 24-hour ambulatory blood pressure monitoring (ABPM), which provides a more accurate reflection of hemodynamic load than office-based measurements. Blood pressure recordings were obtained every 20 min during daytime (06:00–22:00) and every 30 min during nighttime (22:00–06:00). Blood pressure of all participants was measured using a validated electronic sphygmomanometer (Omron HBP-9030) in accordance with standard clinical guidelines. All measurements were performed after the subjects had rested quietly for at least 5 min in a sitting position. Biochemical markers, including lipid profiles, fasting blood glucose, and renal function indices (Creatinine, Urea Nitrogen, eGFR), were measured using standardized laboratory techniques.

### Untargeted metabolomics (UPLC-TOF/MS)

Fasting venous blood samples were collected in EDTA tubes and centrifuged at 3,000 rpm for 10 min at 4 °C to isolate serum. To ensure metabolic stability and prevent enzymatic degradation of labile compounds, samples were stored at −80 °C. For the untargeted phase, serum proteins were precipitated using a chilled water solution. Chromatographic separation was achieved on a Waters ACQUITY BEH C18 column (1.8 µm, 2.1 × 100 mm). Mass detection was performed on an AB TripleTOF 6600 system in both positive and negative electrospray ionization (ESI) modes. Quality control (QC) samples, prepared by pooling aliquots from all subjects, were utilized to ensure system stability and reproducibility throughout the analytical run.

### Targeted metabolic quantification (LC-MS/MS)

Absolute quantification of 32 key metabolites within the tryptophan network was conducted using an independent, high-sensitivity LC-MS/MS platform. Multiple reaction monitoring (MRM) transitions were optimized for each molecule (e.g., 5-HT, Kynurenine, Indole-3-acetic acid) using authentic chemical standards. Data were processed using MultiQuant software, and concentrations were normalized using stable isotope-labeled internal standards, ensuring that the quantitative results were robust and reproducible across different batches.

### Network pharmacology and molecular docking analysis

To elucidate the pharmacological mechanisms of the identified metabolites against hypertension, SMILES strings and InChIKeys were retrieved from the PubChem database and utilized to predict putative targets via the TCMSP platform. The resulting intersection targets were mapped to the STRING database to construct a Protein–Protein Interaction (PPI) network, applying a confidence threshold of ≥0.4. Topological visualization and the construction of an integrated metabolite–target–hypertension multidimensional network was executed using Cytoscape (version 3.9.0). Subsequently, the biological functions and therapeutic signaling pathways were deciphered through Gene Ontology (GO) and Kyoto Encyclopedia of Genes and Genomes (KEGG) enrichment analyses using R software. Significant results were prioritized based on a significance threshold of *q* < 0.05 and ranked by statistical stringency. To validate the binding interactions, three-dimensional protein structures were sourced from the RCSB Protein Data Bank and docked with core active components using AutoDock Vina (version 1.1.2). Following the definition of grid box coordinates for the active sites, the binding affinities were quantified, and the most energetically favorable docking configurations were visualized using PyMOL to characterize the intermolecular binding motifs.

### Statistical analysis

Statistical analyses were conducted using SAS software (version 9.4). Continuous variables were evaluated for normality, those following a normal distribution were represented as mean ± standard deviation (SD), while non-normally distributed data were presented as median [interquartile range (IQR)]. For intergroup comparisons, the independent samples t-test was employed for normally distributed variables, while the Wilcoxon rank-sum test was utilized for non-parametric data. Categorical variables were analyzed using the chi-square test. All statistical analyses were two-tailed, with statistical significance defined as a *P* < 0.05.

## Results

### Demographic characteristics of the study population

This study involved 25 newly diagnosed, treatment-naïve hypertension patients (the hypertension group) and 25 healthy controls with a 1:1 ratio matched for age and sex (Table 1). In the hypertension group, both mean daytime systolic and diastolic blood pressure were significantly higher than those in the healthy control group. Total cholesterol (TC) levels and high-density lipoprotein cholesterol (LDL-C) levels were significantly lower in the hypertensive group compared to controls. Notably, estimated glomerular filtration rate (eGFR) was significantly higher in the hypertensive group. No significant differences were observed between the two groups regarding BMI, triglycerides (TG), or high-density lipoprotein cholesterol (HDL-C), or hemoglobin levels.

**Table 1:** Demographic characteristics of hypertensive patients and healthy controls.

Characteristic	Hypertension patients (*n* = 25)	Healthy control (*n* = 25)	*P* value
Age, years	49.5 ± 9.6	49.5 ± 9.6	1.000
Male, *n* (%)	16 (64.0)	16 (64.0)	1.000
BMI/kg·m-2	25.5 ± 3.7	24.1 ± 3.5	0.195
Daytime Mean SBP, mmHg	139.4 ± 20.7	121.9 ± 12.5	<0.001
Daytime Mean DBP, mmHg	82.4 ± 10.5	73.1 ± 8.9	0.001
TG, mmol/L	2.1 ± 1.3	1.9 ± 1.3	0.580
TC, mmol/L	4.4 ± 1.1	5.1 ± 1.0	0.028
HDL, mmol/L	1.0 ± 0.3	1.4 ± 0.4	<0.001
LDL, mmol/L	3.1 ± 1.0	3.1 ± 0.7	1.000
Hemoglobin, g/L	147.7 ± 12.4	144.8 ± 10.1	0.355
eGFR, mL/ (min·1.73 m²)	126.5 ± 24.8	97.7 ± 20.3	<0.001

BMI, body mass index; SBP, systolic blood pressure; DBP, diastolic blood pressure; TG, triglycerides; TC, total cholesterol; HDL-C, high-density lipoprotein cholesterol; LDL-C, low-density lipoprotein cholesterol; eGFR, estimated glomerular filtration rate.

### Plasma metabolic profiling

**Figure 1:**
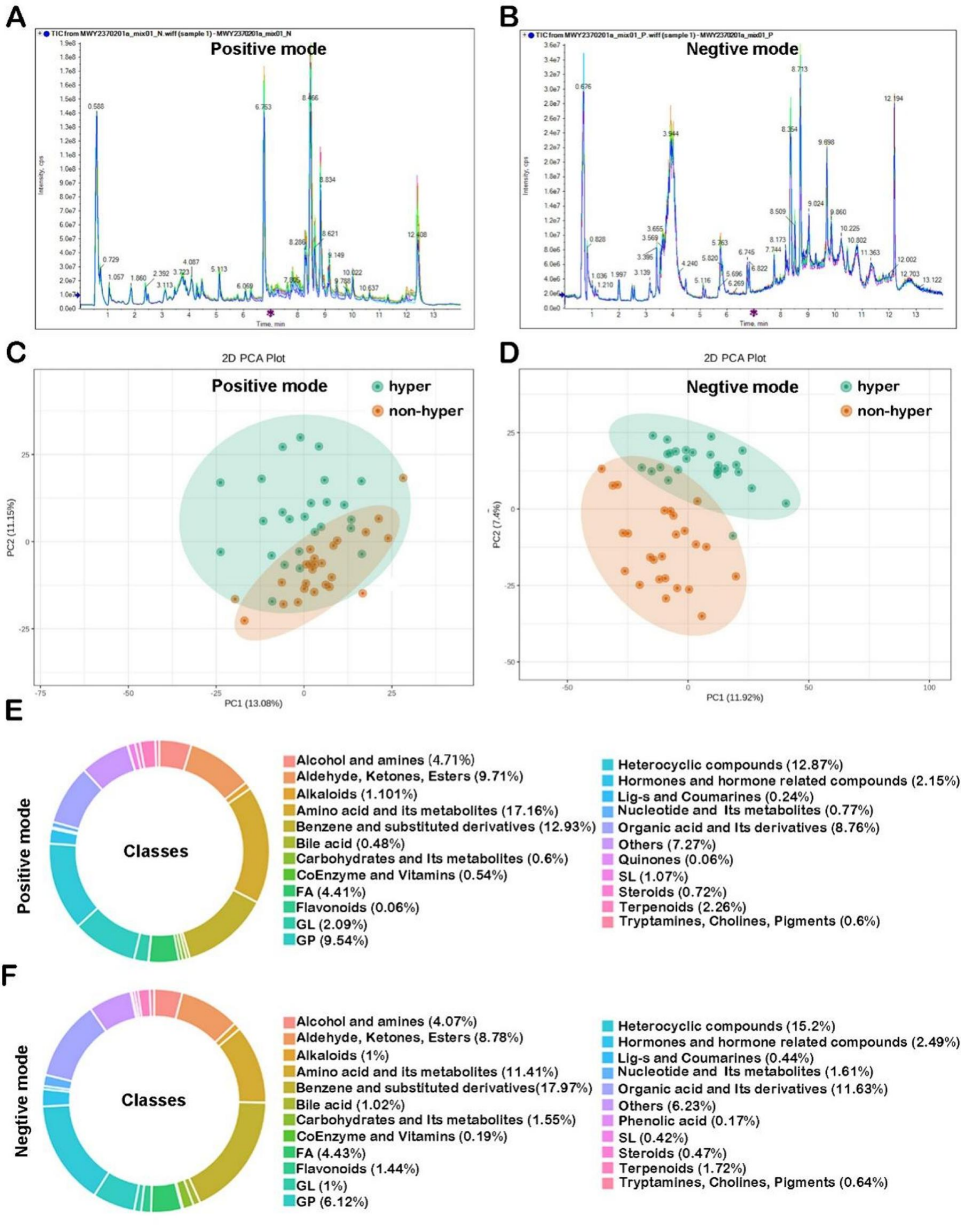
Metabolic profiling of Serum samples. **(A)** Total ion current overlay of QC samples in positive ion mode; **(B)** Total ion current overlay of QC samples in negative ion mode; **(C)** PCA analysis of serum samples from the hypertension and control groups in positive ion mode; **(D)** PCA analysis in negative ion mode; **(E)** Overview of metabolite categories in positive ion mode and **(F)** in negative ion mode for all tested samples.

Total ion chromatograms (TIC) of QC samples obtained via the LC/MS platform are shown in Figures 1A,B. The chromatographic peaks of the QC samples exhibited excellent overlap in both positive and negative ion modes, indicating high stability of the mass spectrometry system. PCA score plots revealed a distinct separation trend between the hypertensive and control groups in both modes, suggesting significant alterations in serum metabolites in hypertensive patients (Figures 1C,D). A total of 2,931 metabolites were identified across the 25 pairs of samples. In positive ion mode, the primary metabolite classes by proportion included amino acids and their derivatives (17.16%), benzene and its substituted derivatives (12.93%), heterocyclic compounds (12.87%), and aldehydes, ketones, and esters (9.71%) (Figure 1E). In negative ion mode, the predominant classes were benzene and substituted derivatives (17.97%), heterocyclic compounds (15.2%), organic acids and derivatives (11.63%), and amino acids and derivatives (11.41%) (Figure 1F). These results demonstrate consistent metabolite categories across both ionization modes.

**Figure 2:**
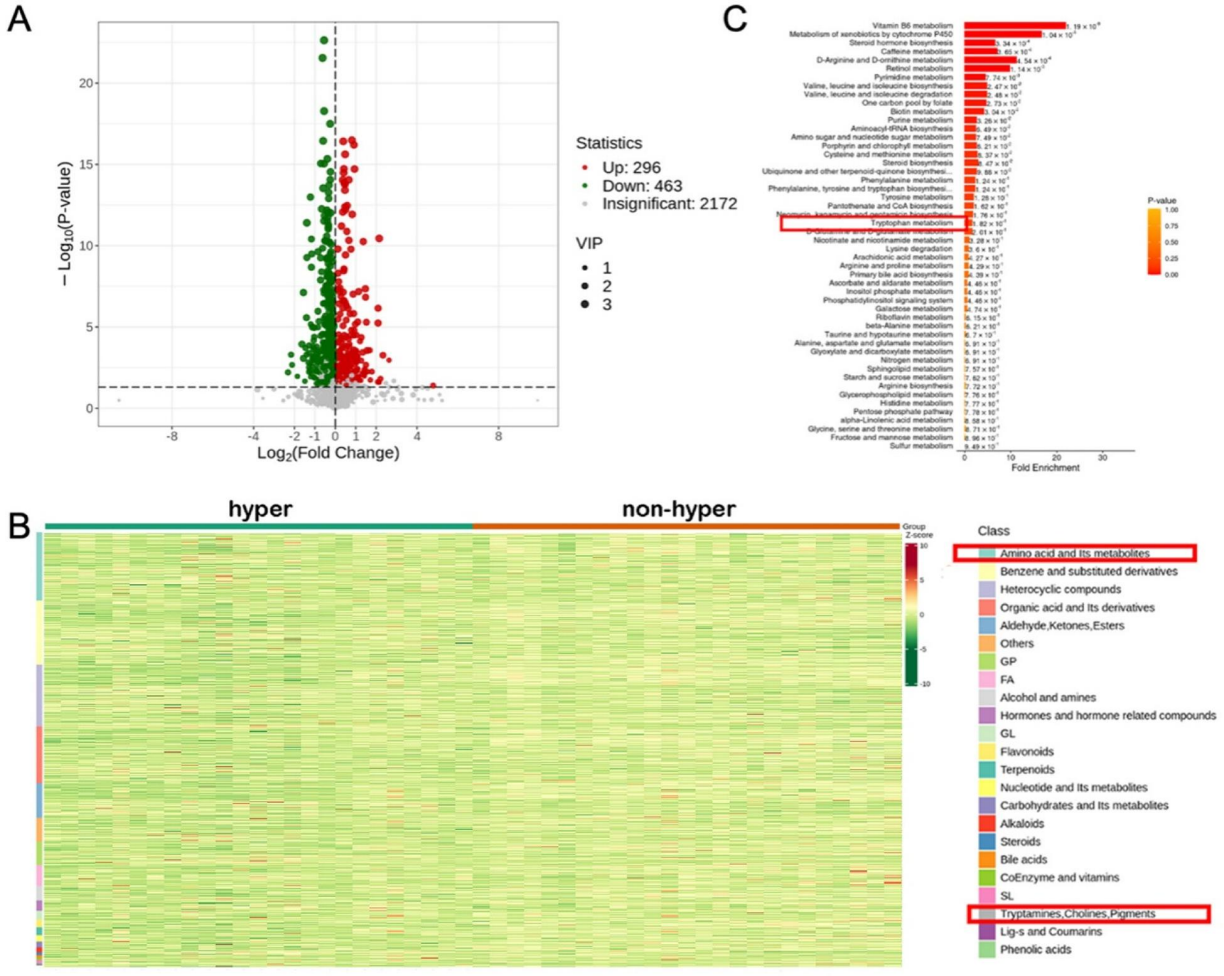
Differential metabolite analysis. **(A)** Volcano Plot, **(B)** Heatmap, and **(C)** GO Analysis.

To further identify for differential metabolites (DMs) in hypertensive patients, a t-test threshold of *P* < 0.05 was applied. A total of 759 DMs were identified between the hypertensive and control groups, comprising 296 upregulated and 463 downregulated metabolites, while 2,172 metabolites showed no significant difference (Figure 2A). Subsequent clustering analysis revealed that amino acid metabolites accounted for the highest proportion, followed by benzene derivatives, heterocyclic compounds, and organic acids/derivatives (Figure 2B). Further analysis of amino acid metabolites indicated enrichment primarily in pathways related to arginine and ornithine; valine, leucine, and isoleucine; cysteine and methionine; tryptophan; and lysine (Figure 2C).

**Figure 3:**
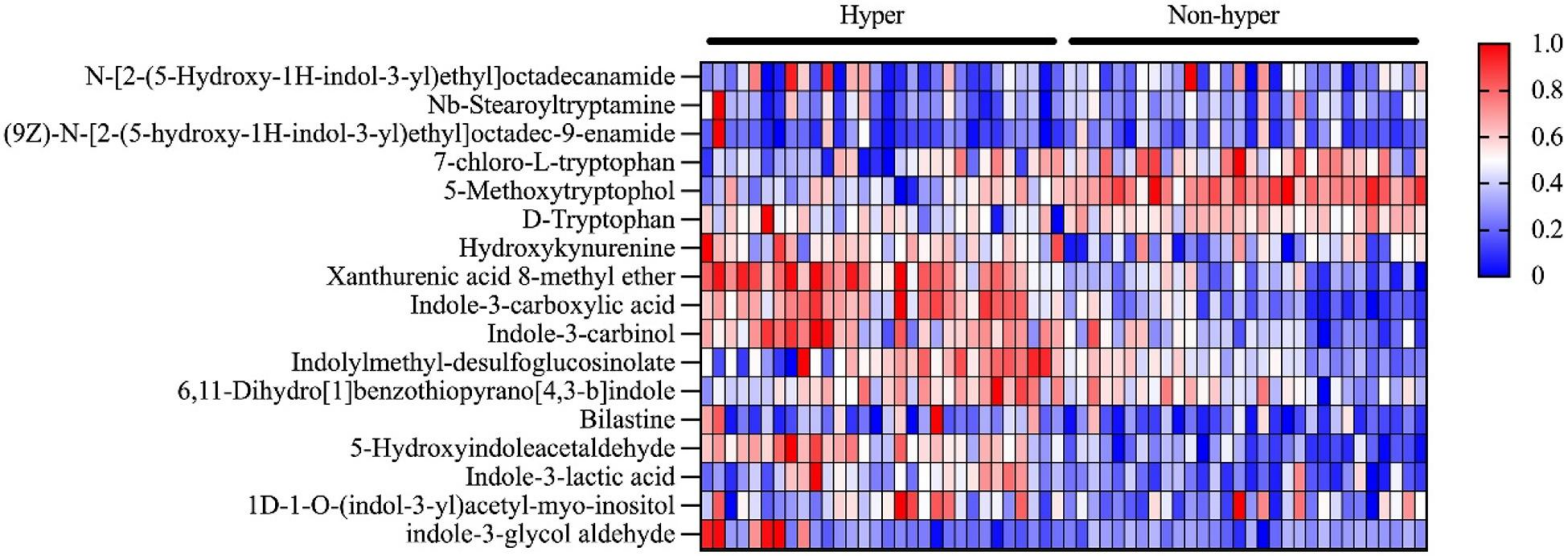
Untargeted metabolomics identifies significantly altered metabolites in the tryptophan metabolic pathway.

Tryptophan is an essential amino acid critical for various physiological functions, including blood-brain barrier integrity and vascular health. As the primary precursor for serotonin (5-HT), tryptophan plays a vital role in regulating vasoconstriction and vasodilation. Serotonin influences blood pressure by modulating peripheral vascular resistance and heart rate. KEGG pathway analysis indicated that three major tryptophan metabolic pathways-indole, 5-HT, and kynurenine-were affected in the hypertensive population. Non-targeted metabolomics identified 17 significantly differential metabolites within the tryptophan pathway: 11 upregulated and 6 downregulated (Figure 3). Specifically, two kynurenine metabolites (8-hydroxykynurenic acid and 8-methoxykynurenate) were upregulated; six tryptamines (oleoyl serotonin, stearoyl serotonin, Nb-stearoyl tryptamine, D-tryptophan, 7-chloro-L-tryptophan, and 5-methoxytryptamine) were downregulated; and nine indole derivatives (e.g., indole-3-carboxylic acid, 3-indolelactate) were upregulated (Figure 3). These findings suggest that hypertension leads to global metabolic shifts in tryptophan and its derivatives, characterized primarily by downregulation of tryptamines and upregulation of kynurenine and indole derivatives.

### Targeted evaluation of serum metabolite variance

**Figure 4:**
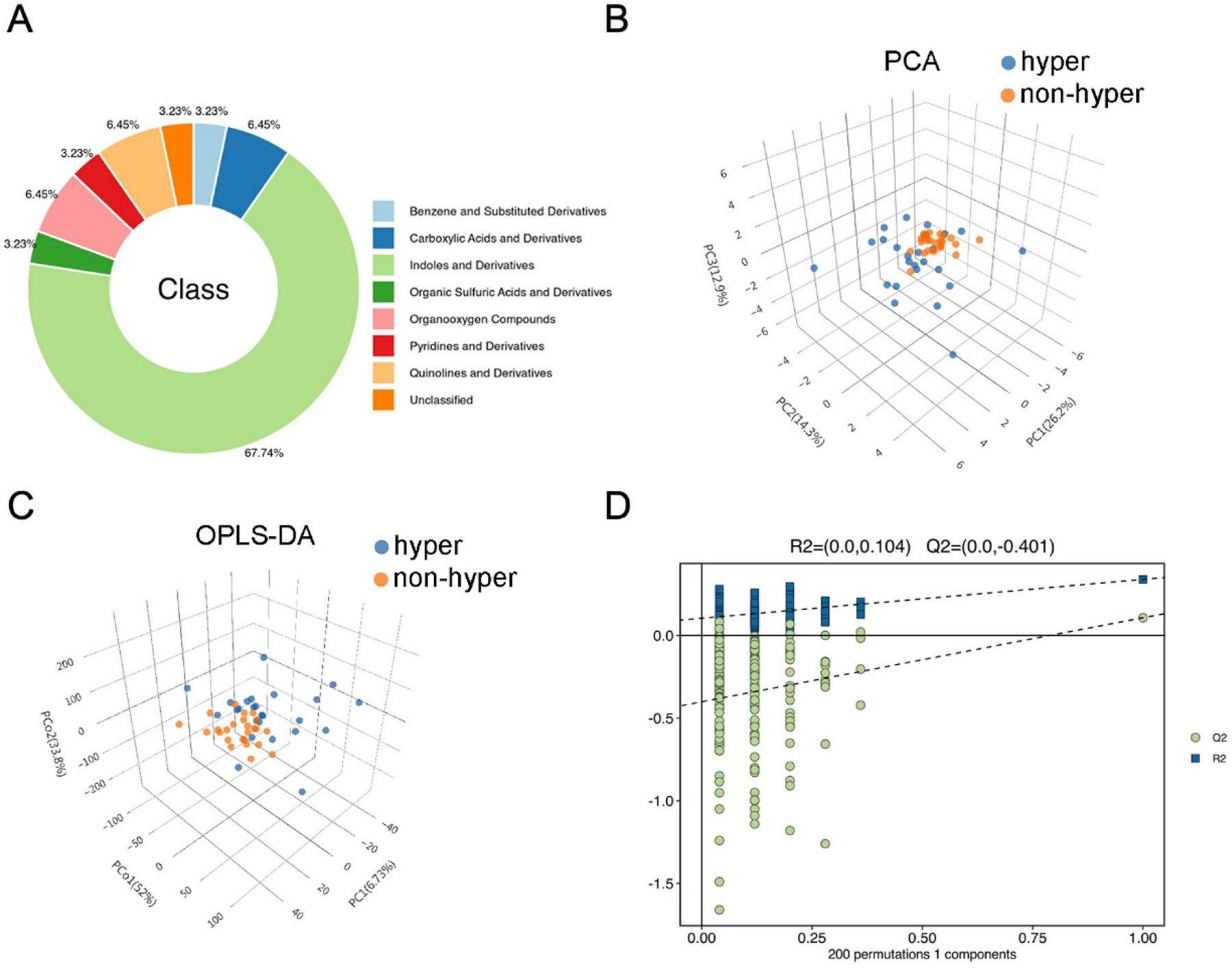
Differential metabolite screening by tryptophan-targeted metabolomics. **(A)** Metabolite Overview; **(B)** PCA Analysis; **(C)** OPLS-DA Analysis; **(D)** Permutation Test.

To further elucidate the relationship between tryptophan metabolism and hypertension, targeted metabolomics was employed for absolute quantification of the three primary pathways. We selected 32 key metabolites covering the serotonin, kynurenine, and indole pathways (standard information provided in Supplementary Table 1). Targeted analysis identified seven major chemical classes, including benzene derivatives, carboxylic acids, indoles, organic acid salts, organic oxygen compounds, pyrimidines, and quinolones (Figure 4A). PCA results showed differentiation between the hypertensive and control groups (Figure 4B). To refine these results and filter out noise, an OPLS-DA model was utilized, which successfully discriminated between the two groups (Figure 4C). Variable importance in projection (VIP) scores were used to measure the influence of each metabolite on group discrimination. Following 200 permutations, the model yielded R^2^Y(cum) = 0.976 and Q^2^(cum) = 0.85, indicating high robustness without overfitting and confirming the biological significance of the identified DMs (Figure 4D).

### Identification of differential serum metabolites

**Figure 5:**
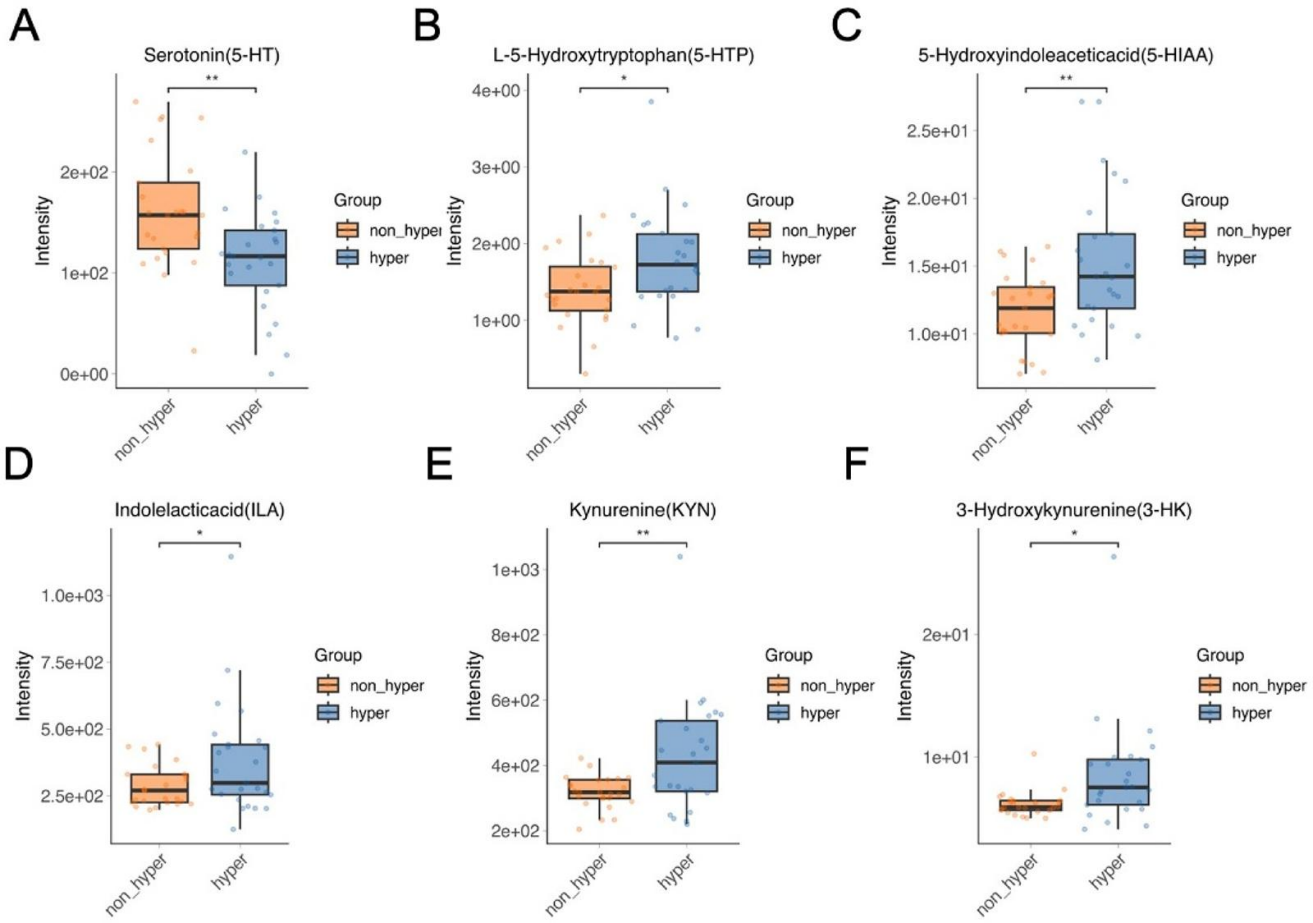
Comparison of tryptophan-related metabolite levels in hypertensive and Non-hypertensive Serum samples. **(A)** Serotonin; **(B)** 5-Hydroxytryptophan; **(C)** 5-Hydroxyindoleacetic Acid; **(D)** Indoleacetic Acid; **(E)** Kynurenine; **(F)** 3-Hydroxykynurenine.

Using criteria of *P* < 0.05 (*t*-test) and a fold change (FC) > 1.2 or <0.7, six significantly differential metabolites were identified: 5-HT, 5-HIAA (5-hydroxyindoleacetic acid), kynurenine (KYN), 3-HK (3-hydroxykynurenine), 5-HTP (5-hydroxytryptophan), and ILA (indolelactate). Compared to the control group, 5-HT levels in hypertensive patients decreased by approximately 30% (Figure 5A). In contrast, levels of the other five metabolites were significantly elevated: 5-HIAA by 33%, KYN by 34%, 3-HK by 38%, 5-HTP by 30%, and ILA by 33% (Figures 5B–F). These metabolites span the three major tryptophan metabolic branches.

**Figure 6:**
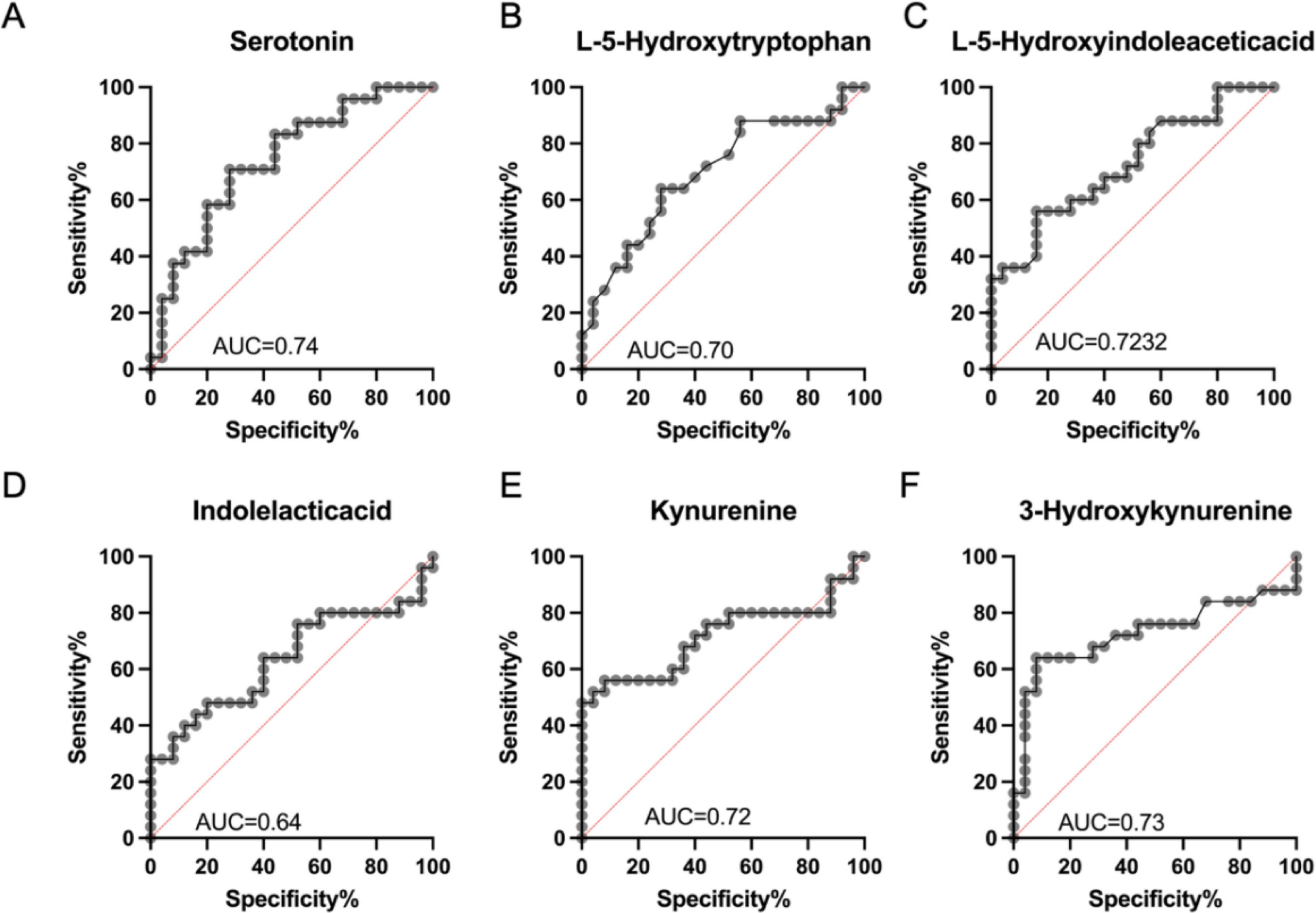
ROC curve analysis of Six hypertension-associated tryptophan metabolites. **(A)** Serotonin; **(B)** 5-Hydroxytryptophan; **(C)** 5-Hydroxyindoleacetic Acid; **(D)** Indoleacetic Acid; **(E)** Kynurenine; **(F)** 3-Hydroxykynurenine (AUC: Area Under the Curve).

Further ROC analysis revealed that the area under the curve (AUC) values for serotonin, 5-hydroxytryptophan, 5-hydroxyindoleacetic acid, kynurenine, and 3-hydroxykynurenine were 0.74, 0.70, 0.72, 0.72, and 0.73, respectively, all exceeding 0.7. This indicates that these metabolites possess potential for predicting hypertension and may serve as biomarkers for hypertension risk assessment. In contrast, the AUC for indoleacetic acid was 0.64, suggesting comparatively limited predictive potential for hypertension (Figure 6).

### Network pharmacology and target prediction

**Figure 7:**
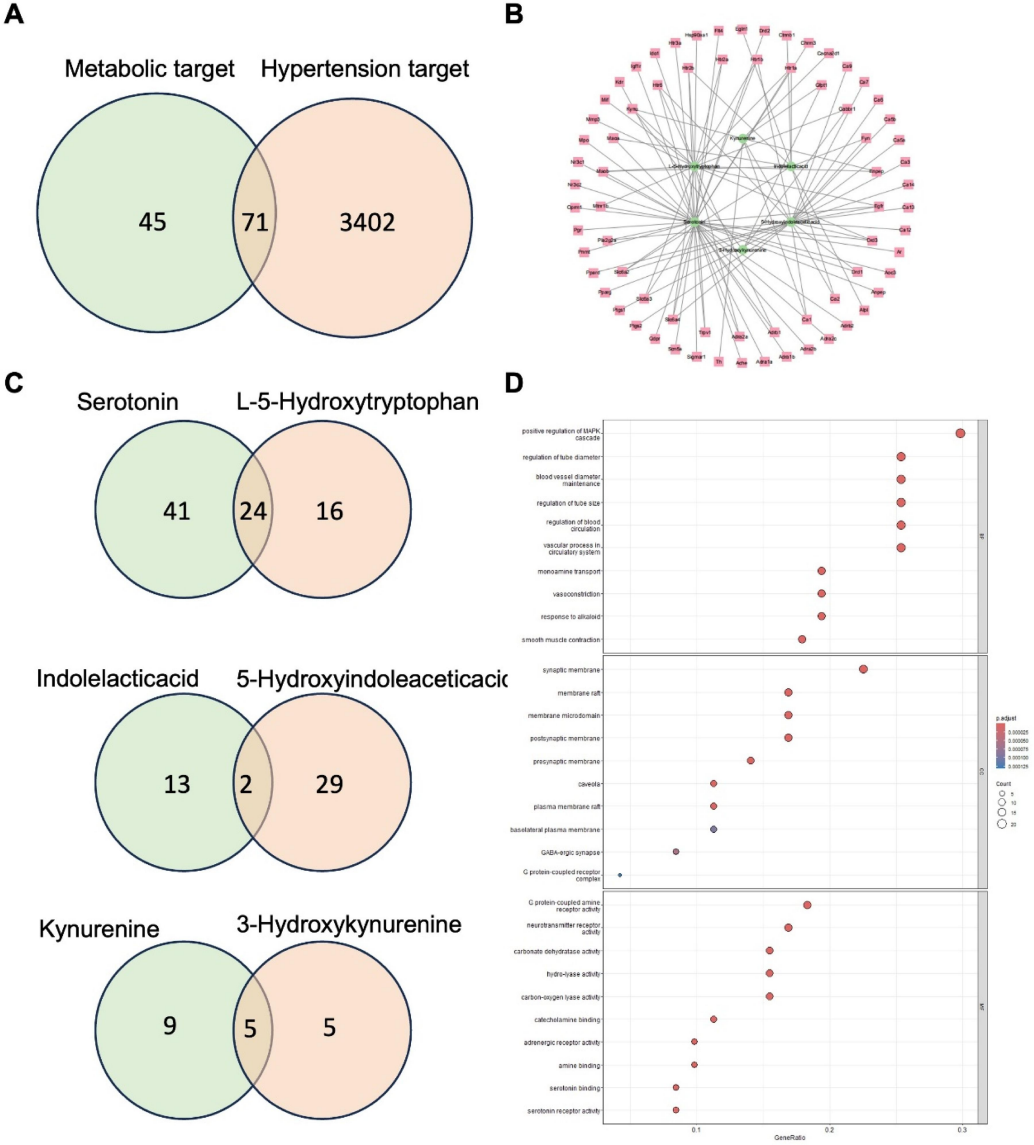
Network pharmacology analysis and target prediction for hypertension biomarkers. **(A)** Venn diagram of the intersecting targets of the six metabolites and hypertension; **(B)** PPI network of the six metabolites and hypertension targets; **(C)** Venn diagrams showing intersections between hypertension targets and targets of tryptamine-, indole-, and kynurenine-class metabolites, respectively; **(D)** GO enrichment analysis of the shared targets.

Metabolite-related targets were retrieved via the TCMSP platform, while hypertension-associated targets were sourced from GeneCards and OMIM databases, totaling 3,518 targets. The intersection of these datasets yielded 71 potential hypertension-related targets for the six metabolites (Figure 7A). Using Cytoscape (v3.10.0), we attempted to identify a single common target across all six metabolites but found no universal intersection (Figure 7B), suggesting that these metabolites regulate blood pressure through distinct target proteins. When grouped by metabolic class, the intersections were as follows: serotonin-related (5-HT and 5-HTP) shared 24 targets; microbial-indoles (5-HIAA and ILA) shared 2 targets; and kynurenines (KYN and 3-HK) shared 5 targets. GO enrichment analysis revealed that these targets are primarily involved in the positive regulation of the MAPK signaling pathway and the modulation of vascular diameter (Biological Process); localized to synapses (Cellular Component); and enriched in functions such as adrenergic receptor activation and tryptamine receptor binding (Molecular Function) (Figures 7C,D).

**Figure 8:**
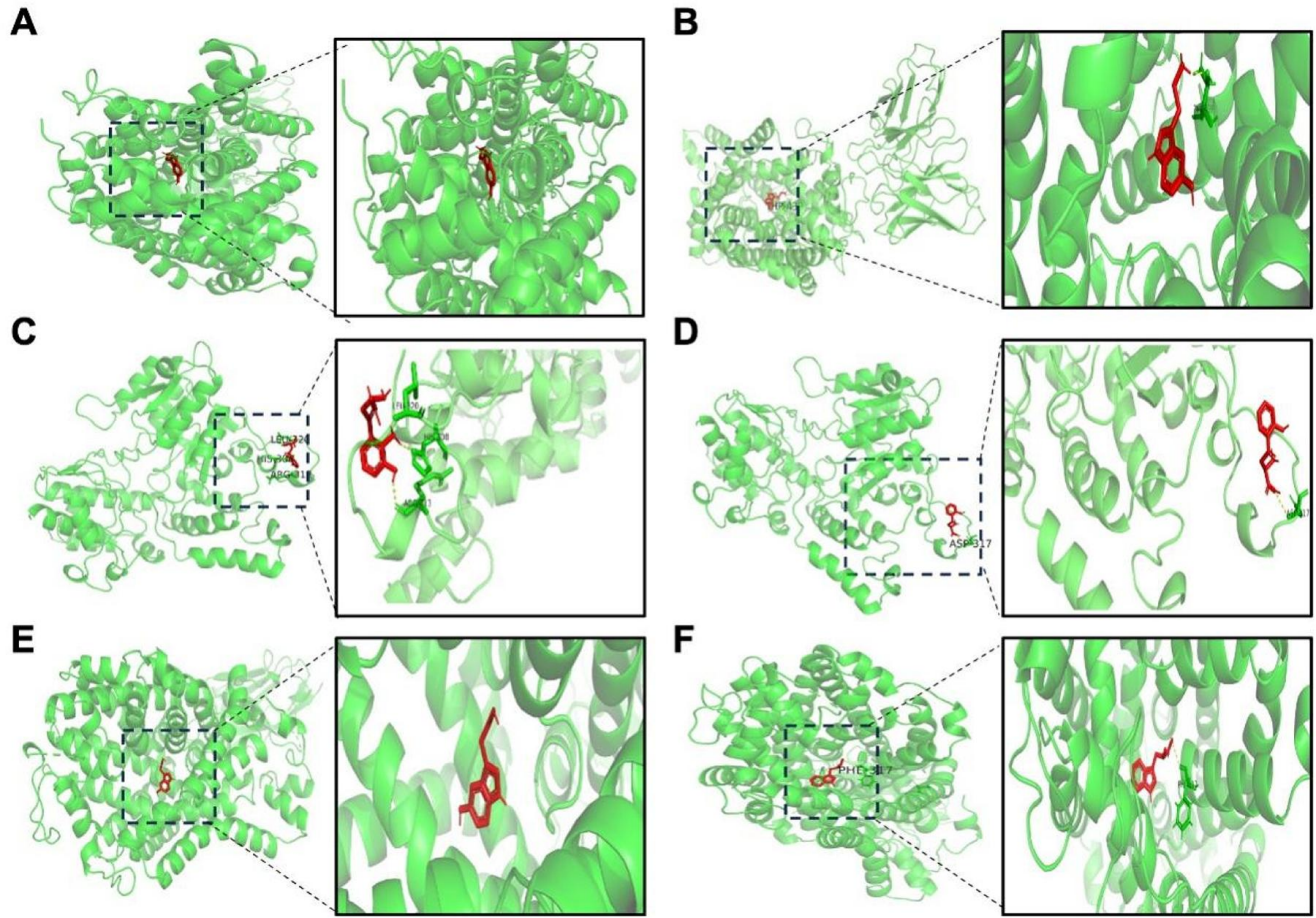
Molecular docking diagrams. **(A)** SLC6A4 with serotonin; **(B)** SLC6A4 with 5-hydroxytryptophan; **(C)** KYNU with 3-hydroxykynurenine; **(D)** KYNU with kynurenine; **(E)** SLC6A2 with 5-hydroxyindoleacetic acid; **(F)** SLC6A2 with 2-indoleacetic acid.

Molecular docking was performed to assess binding affinities. For the serotonin class (Serotonin and L-5-HTP), SLC6A4 was identified as the optimal binding protein based on binding energy and hypertension correlation indices (Figures 8A,B). For the kynurenine class (KYN and 3-HK), KYNU was identified as the best target (Figures 8C,D). For the indole class (5-HIAA and ILA), SLC6A2 emerged as the primary target (Figures 8E,F). Specifically, L-5-HTP binds to SLC6A4 at residues ILE172, GLY442, PHE341, and ASN177; ILA binds to SLC6A2 at ASP75, ALA73, PHE72, TYR152, and VAL148; and 3-HK binds to KYNU at ARG313, MET316, TRP305, HIS308, and LEU320. In summary, SLC6A4, KYNU, and SLC6A2 were identified as the potential core targets for the serotonin, kynurenine, and indole metabolic pathways, respectively.

## Discussion

The metabolic underpinnings of essential hypertension remain a critical frontier in cardiovascular research, as traditional risk factors often fail to account for the complex interplay between host metabolism and systemic pressure regulation. In this study, using a combined approach of non-targeted and targeted metabolomics, we characterized a distinctive metabolic signature in hypertensive patients, centered on the profound dysregulation of tryptophan metabolism. Our findings reveal a systematic redirection of tryptophan flux, characterized by the depletion of the serotonin pathway and concomitant activation of the kynurenine and microbial-indole branches. These metabolic shifts are not merely peripheral markers but are intricately linked to vascular homeostasis, inflammatory signaling, and potentially early-stage renal hyperfiltration, as evidenced by the elevated eGFR observed in our data.

A primary finding of this study is the significant reduction in serum 5-HT levels alongside an accumulation of its precursor, 5-HTP. Tryptophan hydroxylase (TPH) is the rate-limiting enzyme in the conversion of tryptophan to 5-HTP, which is then rapidly decarboxylated to 5-HT by aromatic L-amino acid decarboxylase (AADC) or, more likely, systemic shunting of Trp away from the serotonin branch toward competing pathways (14, 15). Peripherally, 5-HT is a complex modulator of vascular tone, capable of inducing both vasoconstriction and vasodilation depending on the specific receptor subtypes and the pre-existing state of vascular contraction (16, 17). Chronic depletion of circulating 5-HT may disrupt this delicate balance, contributing to the elevated systemic vascular resistance characteristic of hypertension (18, 19). SLC6A4 (serotonin transporter, SERT) was found to be a high-affinity binding target for 5-HT metabolites. The dysregulation of serotonin transport may be correlated with metabolic remodeling and impaired vascular regulation in patients with hypertension.

Complementing the decrease in serotonin signaling is the marked upregulation of the kynurenine pathway, evidenced by the elevated levels of KYN and 3-HK. Under physiological conditions, the majority of dietary tryptophan is processed through the kynurenine pathway, regulated by the rate-limiting enzymes IDO and TDO (20, 21). In the context of hypertension, low-grade chronic inflammation often stimulates IDO activity via pro-inflammatory cytokines like IFN-*γ*. The subsequent accumulation of 3-HK is of particular concern, as it is a known pro-oxidant capable of generating ROS (22, 23). Increased ROS generation in the endothelium enhances NO consumption, resulting in endothelial impairment and heightened arterial rigidity. Our network pharmacology investigation pinpointed KYNU as a key player, reinforcing this process. KYNU, accountable for metabolizing kynurenine derivatives into anthranilic acid and 3-hydroxyanthranilic acid, interacting with KYN and 3-HK, implies that the KYN-KYNU axis could serve as a pivotal juncture in the vascular inflammatory pathway in hypertension advancement.

Another striking observation is the significant increase in microbial-derived indole derivatives, specifically ILA and 5-HIAA. These metabolites are products of tryptophan fermentation by the gut microbiota, highlighting a potential gut-vessel axis in blood pressure regulation. Indole derivatives are endogenous ligands for the aryl hydrocarbon receptor, which plays a multifaceted role in immune modulation and vascular integrity. Elevated ILA suggests a compensatory or maladaptive response of the gut microbiome to the hypertensive environment (24). Furthermore, the docking analysis pinpointed SLC6A2 (norepinephrine transporter, NET) as the optimal target for these indole metabolites (25). The cross-talk between microbial indoles and catecholamine transporters like SLC6A2 presents a novel hypothesis: that gut-derived metabolites may influence blood pressure by modulating the reuptake of norepinephrine, thereby affecting sympathetic nervous system activity (26, 27). This integration of microbial metabolism with autonomic regulation offers a new perspective on the systemic nature of hypertension.

The network pharmacology and GO enrichment analysis provided mechanistic depth to our metabolic findings, identifying the MAPK signaling pathway as a primary downstream effector. The MAPK cascade is a central regulator of VSMC proliferation, migration, and contraction (28). Our results suggest that the altered Trp metabolites do not act in isolation but converge on signaling hubs that control vascular diameter and structural remodeling. The localization of these targets to synaptic components and their involvement in adrenergic receptor activation further bridge the gap between metabolic flux and the physiological control of heart rate and peripheral resistance.

Notably, we observed markedly higher eGFR levels in the hypertensive group relative to controls, which is an intriguing and unexpected result. Several underlying factors may jointly account for this phenomenon. First, glomerular hyperfiltration is a well-recognized pathological feature of early-stage hypertension. Increased systemic blood pressure can raise intraglomerular pressure, leading to compensatory elevation of glomerular filtration rate before the occurrence of overt renal impairment. Second, our study mainly enrolled outpatients with newly diagnosed or mild hypertension without severe target organ damage. This inclusion characteristic indicates a potential selection bias, as our cohort was predominantly composed of patients in the early phase of hypertension rather than those with long-standing disease and progressive renal dysfunction. Combined with our metabolomic results, this renal functional change is closely correlated with disturbed tryptophan metabolism, especially the overactivated kynurenine pathway. Excessive accumulation of kynurenine and its metabolites may act on renal microvasculature and participate in the regulation of glomerular hemodynamics. This evidence further supports the existence of the tryptophan-renal axis in hypertension (29). The lower total cholesterol and HDL-C levels in the hypertensive cohort were unexpected but may reflect a specific metabolic phenotype or the influence of prior dietary not fully captured at baseline (30, 31). Nevertheless, the robustness of our metabolomic data, validated across both non-targeted and targeted platforms, ensures that the identified Trp metabolic shift is a core feature of the study population.

Our study had limitations. First, the relatively small sample size may compromise the statistical robustness and generalizability of the untargeted metabolomics results, and insufficient statistical power cannot be fully ruled out when screening differential metabolites. Second, while the study validates the central role of the tryptophan network using an integrated omics approach, its cross-sectional design precludes definitive causal inferences regarding the temporal relationship between metabolic shifts and blood pressure elevation. Future longitudinal cohorts and mechanistic studies are warranted to further elucidate the downstream signaling pathways by which specific metabolites modulate vascular smooth muscle function.

In conclusion, this study identifies specific alterations in tryptophan metabolism as a hallmark of hypertension. The results show a significant decrease in serotonin levels alongside an increase in kynurenine and indole derivatives. SLC6A4, KYNU, and SLC6A2 were identified as key molecules correlated with blood pressure regulation, which may exert effects via the MAPK signaling pathway. These findings suggest that the tryptophan-serotonin-kynurenine metabolic axis provides a basis for developing new diagnostic biomarkers for hypertension.

## Data Availability

All omics data and de-identified researcher data generated in this study are available from the corresponding author (Zhen-xian Zhang) upon reasonable request via email.
